# Ethanol Extract of the Infructescence of *Platycarya strobilacea* Sieb. et Zucc. Induces Methuosis of Human Nasopharyngeal Carcinoma Cells

**DOI:** 10.1155/2020/2760979

**Published:** 2020-04-29

**Authors:** Jinkun Liu, Min Ying, Bin Wu, Chaomei Fu

**Affiliations:** ^1^College of Pharmacy, Chengdu University of Traditional Chinese Medicine, Chengdu 611137, China; ^2^Chongqing Key Laboratory of Translational Research for Cancer Metastasis and Individualized Treatment, Chongqing University Cancer Hospital, Chongqing 400030, China; ^3^Chongqing Key Laboratory of Traditional Chinese Medicine to Prevent and Treat Autoimmune Diseases, Chongqing Traditional Chinese Medicine Hospital, Chongqing 400021, China

## Abstract

The infructescence of *Platycarya strobilacea* Sieb. et Zucc. (PS) has been used in the treatment of rhinitis and sinusitis in clinical practice. Our preliminary study showed that an ethanol extract of the infructescence of PS (EPS) had significant antinasopharyngeal carcinoma (NPC) effects in vitro. However, the mechanism underlying the NPS cell death induced by EPS remains unclear. The aim of the present study was to investigate the inhibitory effects of EPS on NPC cells and to elucidate the underlying mechanism. The effects of EPS on NPC cells were investigated in CNE1 and CNE2 cells in vitro. In EPS-treated cells, the cell morphological changes were evaluated through fluorescence microscope, transmission electron microscopy, and flow cytometry. The underlying mechanism was analyzed via network pharmacology and further verified by western blot analysis. The anticancer effects of EPS were associated with the generation of CNE1 and CNE2 cell fusion and vacuoles, the perturbation of lysosomal vesicle transportation, and the induction of methuosis. The network pharmacology and western blot results indicated that the effect of EPS in NPC cells might be achieved via regulation of the Ras proto-oncogene (RAS)/mitogen-activated protein kinase (MAPK) signaling pathway and the transcription factor c-Fos proto-oncogene (c-FOS) and its downstream genes. EPS induces NPC cell death through methuosis. The mechanism might be related to regulation of the transcription factor c-FOS and its downstream genes.

## 1. Introduction

Nasopharyngeal carcinoma (NPC) is a malignant tumor derived from human nasopharyngeal epithelial tissue. One report estimated that 129,079 new cases of NPC and 72,987 NPC-related deaths occurred worldwide in 2018 [1]. The number of NPC patients diagnosed in China within the last 5 years reached 138,500 [2]. At present, the clinical treatment of NPC is mainly based on radiotherapy supplemented by chemotherapy, and no specific drugs for this disease are available [3]. Therefore, identification of new therapeutic targets for drugs, which will help improve the cure and survival rates of NPC and enhance patient quality of life, is important.

*Platycarya strobilacea* Sieb. et Zucc. (PS) has been widely recognized as a medicinal plant from China with various beneficial effects. The infructescence of PS is believed to eliminate toxic heat, activate blood circulation, relieve swelling, eliminate pus, and ameliorate pain [4–6]; it has also been used in NPC treatment [7]. The Chinese herbal medicine Xiangju capsule, which includes this infructescence as its main component, has been applied in the clinical treatment of rhinitis and sinusitis for more than 20 years. This treatment can induce human leukocytes to produce interferon and improve immunity [8]. The main constituents identified from this infructescence are polyphenols, ellagitannins, and flavone-related compounds [9]. These components include ellagic acid, gallic acid, and ursolic acid, which have antioxidative and anti-inflammatory effects [10, 11]. Our previous experimental study found that ethanol extract of PS (EPS) induced CNE1 and CNE2 cell death, which was similar to methuosis. Methuosis is a form of cell death that ultimately leads to rupture through the production of many intracellular vesicles [12, 13].

However, to our knowledge, the antitumor properties of EPS have not been investigated. We conducted the present research to investigate the inhibitory effect of EPS on NPC cells and to elucidate the intracellular pharmacological mechanism.

## 2. Materials and Methods

### 2.1. Plant Material

The infructescence of PS was collected in August of 2016 in the vicinity of Dayuanzi Village, Qikou Town, Lueyang County, Hanzhong City, Shanxi Province, China (position: latitude 33.183675°, longitude 106.358065°).

Plant material (4500 g) with the seeds removed was smashed with a 60 mesh sieve. Powder was extracted with 13500 mL of 95% (v/v) ethanol in a shaker bath set at 30°C for 0.5 h, and this process was repeated three times. Ethanol was removed from the combined filtrate at 45°C using a rotary evaporator. A total of 180 g of extract was obtained after the aqueous phase, and the yield was 4.5%. A voucher specimen (No. 20160801) was deposited in the Chinese medicine preparation laboratory. HPLC was used to identify the active ingredients in the EPS (Supplemental Table 1).

### 2.2. Chemicals and Reagents

Methyl thiazolyl tetrazolium (MTT) was purchased from Sigma (Sigma-Aldrich, Inc., St Louis, Missouri, USA). LysoTracker Green DND-26 (L7526) and Hoechst 33342 (R37605) were purchased from Invitrogen (Life Technologies, Shanghai, China). An Annexin V-FITC Apoptosis Kit (556547) and a Cell Cycle Detection Kit (340242) were purchased from BD (BD Biosciences, Inc., San Jose, California, USA). The following antibodies were used in this work: HRas proto-oncogene (HRAS, ab32417), Rac family small GTPase 1 (RAC1, ab155938), coiled-coil domain-containing protein 42 (CDC42, ab187643), Raf-6 proto-oncogene (RAF6, ab131261), member of the RAS oncogene family 7 (RAB7, ab137029), Ras homolog gene family member A (RhoA, ab187027) (Abcam, Cambridge, UK), extracellular regulated protein kinase 1/2 (ERK1/2, 137F5, 4695), caspase-3 and cleaved caspase-3 (D3R6Y, 14220), phosphorylated ERK1/2 (p-ERK1/2, Thr202/Tyr204, 4370), c-Fos proto-oncogene (c-FOS, 9F6, 2250; Cell Signaling Technology, Boston, Massachusetts, USA), and glyceraldehyde-3-phosphate dehydrogenase (GAPDH, 10494-1-AP). Horseradish peroxidase-labeled secondary antibodies (SA00001-2; Proteintech Group, Inc., Chicago, Illinois, USA) were also used. *β*-sitosterol (22564) and EHT 1864 (HY-16659, an inhibitor of RAC1) were purchased from MedChemExpress (Monmouth Junction, New Jersey, USA). EHT 1864 was dissolved in dimethyl sulfoxide (DMSO) at 10 mM and stored at −20°C. Gallic acid (110831–201605), ellagic acid (111959–201602), and ursolic acid (110742–201421) were obtained from the National Institutes for Food and Drug Control (Beijing, China).

### 2.3. Cell Culture

The CNE1 and CNE2 cell lines were obtained from the Cell Bank of the Chinese Academy of Science (Shanghai, China). The NP69 cell line was provided by the University of Hong Kong. The cells were maintained in RPMI 1640 medium supplemented with 10% fetal bovine serum (FBS) (Biological Industries, Inc., Kibbutz Beit Haemek, Israel) at 37°C in a humidified chamber containing 5% CO_2_.

### 2.4. Cell Viability Assays

Cell viability was quantified by MTT assays. CNE1 and CNE2 cells were plated at a density of 2.5 × 10^3^ cells/well in 96-well plates with eight replicates for each condition. After 24 h, various concentrations of EPS (0, 2.5, 5, 10, 15, 20, 30, and 50 mg/mL) were added to each well, and the plates were incubated for 24 h. Then, 5 mg/mL of MTT solution was added at 20 *µ*L/well and incubated for 4 h at 37°C. At the end of the incubation, the MTT-formazan was solubilized in DMSO, and the absorbance was determined with a 96-well plate reader at 490 nm.

### 2.5. Apoptosis Assay

The cancer cells were seeded into six-well plates at a density of 2.5 × 10^5^ cells/well and then treated with EPS (1.0 mg/mL). After 24 h of incubation, apoptosis was evaluated using an Annexin V-Fluorescein Isothiocyanate (Annexin V-FITC) Kit following the manufacturer's instructions.

### 2.6. Cell Cycle Analysis

The cancer cells were seeded into 10 cm plates in 1.0 mg/mL EPS and RPMI 1640 containing 10% FBS. After 24 h, 5 × 10^6^ cells were harvested, rinsed with cold PBS, and fixed with 75% ice-cold ethanol for 24 h at 4°C. Then, the fixed cells were washed with cold PBS and incubated with propidium iodide (PI, 10 *µ*g/mL) and RNase A (0.5 mg/mL) for 30 min at 37°C. The procedure was the same for the control group and the experimental group after the cells were recultured in complete culture medium. The DNA content of the labeled cells was quantified by FlowJo 7.6.1 software.

### 2.7. Transient Transfection with the RAC1 Inhibitor EHT 1864

Twenty-four hours before transfection, the CNE1 and CNE2 NPC cells were plated onto 10 cm plates at 50% confluence. EHT 1864 at 40 *µ*M was added to the cells for 24 h, and the protein levels were then detected by western blot analysis.

### 2.8. Live Cell Imaging with Fluorescent Tracers

CNE1 and CNE2 cells were seeded in 6-well plates at a density of 2 × 10^5^ cells/well. Labeling of intracellular acidic compartments with LysoTracker Green DND-26 and staining for EPS activity were performed as described previously. Hoechst 33342 (50 *μ*g/mL) was used to label the nucleus for 15 min at 37°C with protection from light. Fluorescence intensity was quantified by ImageJ.

### 2.9. Transmission Electron Microscopy (TEM)

Human CNE1 and CNE2 cells were exposed to 1.0 mg/mL EPS for 24 h, treated with 2% citrate fixative, dehydrated with different concentrations of ethanol, infiltrated with acetone, sliced after embedding, double stained with uranyl acetate and lead citrate, and examined with H-7650B TEM (Hitachi, Hitachinaka, Japan).

### 2.10. Pathway Enrichment Analysis

Gene ontology (GO) functional and pathway enrichment analyses of differentially expressed genes (DEGs) were carried out using the clusterProfiler package of *R* [14]. The GO terms and pathway terms with adjusted *P* values <0.05 were selected. The search tool for the retrieval of interacting compounds/proteins in the traditional Chinese medicine systems pharmacology database and analysis platform (TCMSP, version 2.3) was used for the prediction of compound-protein interaction information [15]. We set the criteria of oral bioavailability (OB) greater than 40% and drug-likeness (DL) greater than 0.18.

### 2.11. Western Blot Analysis

The cells were lysed in RIPA buffer (70-WB020, MultiSciences, Hangzhou, China). The protein concentration was detected by bicinchoninic acid assays (5000002, Bio-Rad, Hercules, California, USA). The total protein concentration was determined, and the protein samples were separated by SDS-polyacrylamide gel electrophoresis. Nonspecific binding was blocked with 3% bovine serum albumin in Tris-buffered saline (pH 7.5); subsequently, the membranes were incubated with primary antibodies at 4°C and then with the corresponding horseradish peroxidase-labeled secondary antibodies. The immunoreactive bands were detected using an enhanced chemiluminescent detection (ECL) kit (70-P1425, MultiSciences, Hangzhou, China).

### 2.12. Statistical Analyses

The experiments presented are representative of at least three independent repetitions. All data were analyzed using *R* (3.6.1) and SPSS 20.0 software. The data are presented as the mean ± SD. A two-tailed Student's *t*-test was used for comparisons of the means of two independent groups. ANOVA was used for comparisons of more than two groups. *P* < 0.05 was considered significant.

## 3. Results

### 3.1. EPS Inhibited NPC Cell Viability

**Figure 1:**
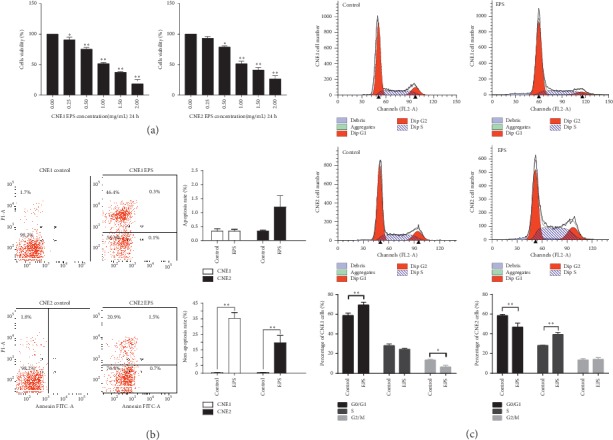
Effects of EPS on NPC cell viability and the cell cycle. (a) CNE1 and CNE2 cells were treated with various concentrations (0–2.0 mg/mL) of EPS for 24 h. Cell viability was detected using an MTT assay. (b) NPC cells were subjected to 1.0 mg/mL EPS for 24 h and then assessed for apoptotic cell death by flow cytometry. The total percentages of apoptotic cell death and nonapoptotic cell death were calculated, as described in the methods. (c) Cell cycle analysis of CNE1 and CNE2 cells treated with the control and 1.0 mg/mL EPS for 24 h. Representative histograms show the distribution of cells in the cell cycle for each treatment group. Data are expressed as the mean ± SD of three independent experiments, ^*∗*^*P* < 0.05, ^*∗∗*^*P* < 0.01 vs the control group, as analyzed by Student's *t*-test.

To examine the effect of EPS on cell proliferation, we treated CNE1 and CNE2 cells with multiple concentrations of EPS. As shown in Figure 1(a), the group treated with the lowest concentration of EPS (0.5 mg/mL) showed a significant difference compared with the control group (*P* < 0.01). The IC50 values of the CNE1 and CNE2 cell lines for EPS were 1.025 and 1.109 mg/mL, respectively. NPC cell viability decreased as the EPS concentration increased. A concentration (1.0 mg/mL) that showed a moderate antiproliferative effect was used for the subsequent experiments.

Next, the EPS-induced loss of cell viability was confirmed using an Annexin V-FITC/PI-binding assay. Compared with the control cells, EPS-treated cells did not show an increased apoptotic fraction, indicating that EPS suppressed cell proliferation by other cell death mechanisms, Figure 1(b).

### 3.2. EPS Regulated the Cell Cycle

As shown in Figure 1(c), after treatment with 1.0 mg/mL EPS for 24 h, the cell population in the *G*0/*G*1 phase increased to 71.15% for CNE1 cells and decreased to 43.86% for CNE2 cells compared with that of the control group. These data indicated that EPS blocked the cell cycle transition from the *G*1 phase to the *S* phase in the CNE1 cell line and the *G*2 phase in the CNE2 cell line.

### 3.3. EPS-Induced Cell Methuosis

**Figure 2:**
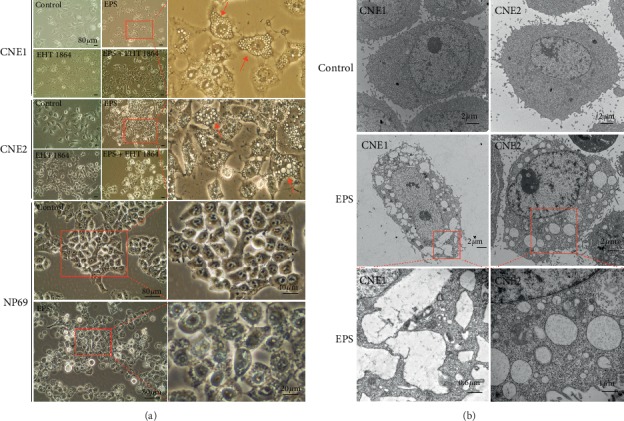
Cell phenotypic changes after NPC cells and NP69 cells were incubated with 1.0 mg/mL EPS and 40 *μ*M EHT 1840 for 24 h. (a) Microscopic analysis of EPS-treated NPC cells and NP69 cells. (b) CNE1 and CNE2 cells were observed by TEM.

Interestingly, all of the cells treated with EPS (1.0 mg/mL) for 24 h showed methuosis-like features [16], such as the formation of multiple protrusions in the cell membrane, and the protrusions between the cells were constantly in contact. Many vesicles were produced in the cells, and the multiple vesicles fused into larger vesicles. This change led to extensive cellular vacuolization and ultimately cell death. EHT 1864 is a small molecule inhibitor of RAC1 signaling. This inhibitor did not prevent EPS-induced cell vesicle production. However, this phenomenon was not observed in normal human epithelial NP69 cells given the same treatment (Figure 2(a)).

TEM analysis of cells treated with EPS showed that the cell membranes budded to form bulges, and the bulges formed multiple small vesicles, which then coalesced into a larger structure; these changes resulted in cell membrane rupture (Figure 2(b)). The transparent vesicles in the cells have a single layer membrane, do not contain cytoplasmic components or organelles, and are distinct from the autophagosomes surrounded by a bilayer membrane formed by autophagy. The morphological characteristics of the dying cells were inconsistent with those of various forms of cell death, such as apoptosis, autophagy, and pyroptosis. The TEM results were consistent with the findings of cells undergoing methuosis.

**Figure 3:**
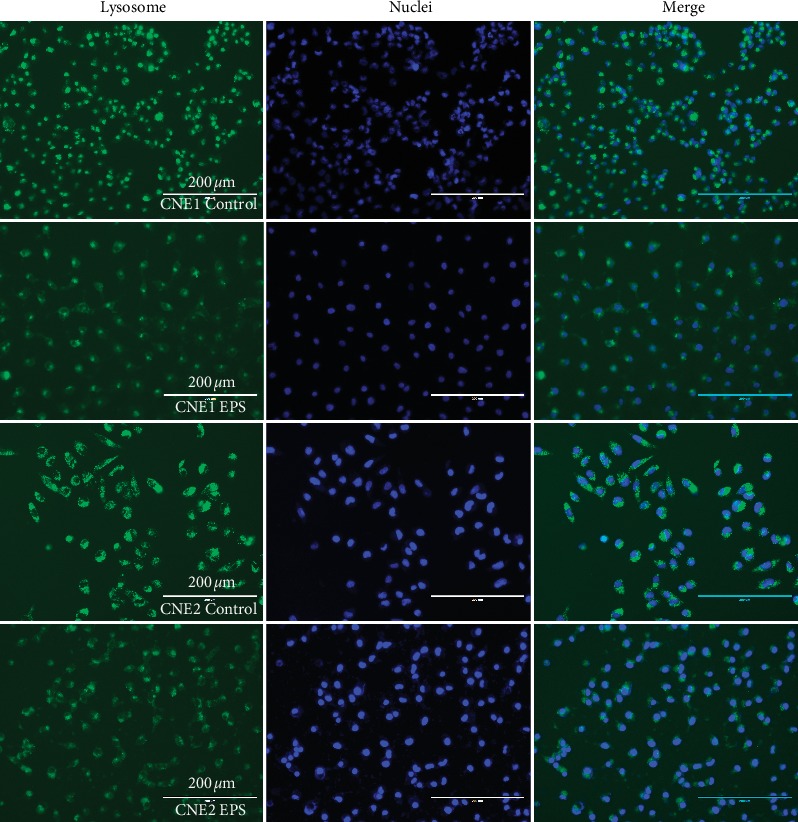
Lysosomes profile in CNE1 and CNE2 after 24 h exposure to EPS (1.0 mg/mL). Lysosomes (green) staining was done with LysoTracker Green DND-26, and nuclei (blue) were counterstained with Hoechst 33342 (50 *μ*g/mL) for 15 min at 37°C.

Then, we tested the relationship between lysosomes and methuosis. Lysosomes were visualized by LysoTracker Green DND-26. We found that, in the EPS group, the number and fluorescence intensity of cells were reduced. Intensity quantification revealed that EPS groups have about 29% and 41% reduction of LysoTracker Green DND-26 staining, respectively, compared to control groups (Figure 3). LysoTracker Green DND-26's fluorescence decreases in alkaline environments, respectively. The results suggested that the pH value of cells increased after adding EPS.

### 3.4. Expression Profiles of NPC Cells after EPS Treatment

**Figure 4:**
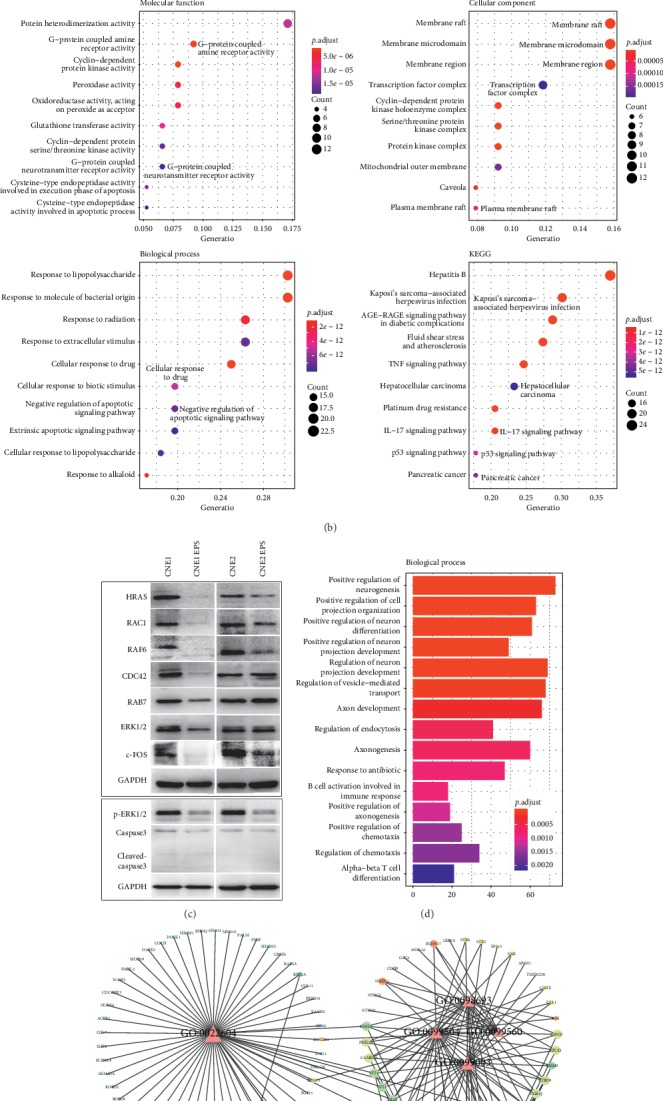
The mechanisms of methuosis induction by EPS in NPC cells, as shown by network pharmacology. (a) Construction of the network map between the active ingredients of EPS and their associated targets. The network was constructed with five candidate compounds and their putative targets, which were constituents of EPS. The green nodes represent candidate compounds, and the targets are indicated by pink circles. (b) GO terms and pathways enriched in the network targets of EPS. The dot size indicates the ratio of genes in the respective pathways to the total number of tested genes, and the dot color illustrates the adjusted *P* value of the enrichment. (c) Immunoblot analysis of senescence-related proteins in EPS-treated NPC cells. GAPDH was used as a control for these proteins. (d) The GO terms in the biological process categories at all levels enriched in downstream target genes of c-FOS. (e) Construction of the GO BP terms. The histogram was generated by connecting the candidate GO terms and their targeted genes, which were related to methuosis.

To identify the potential genes affected by EPS treatment, we combined OB screening and DL evaluation to identify the active compounds in EPS, as shown in Figure 3(a). Five compounds (ursolic acid, ellagic acid, gallic acid, 3,3′-dimethoxy ellagic acid, and beta-sitosterol) were widely distributed in EPS. The contents of gallic acid, *β*-sitosterol, ellagic acid, and ursolic acid in EPS were 0.72%, 1.85%, 4.99%, and 1.88%, respectively (Supplemental Figure 1). The total content of the four components in the EPS was 9.44%. We screened 109 candidate targets by TCMSP, explored the biological networks, and analyzed the relationships between the functional groups (Figure 4(a)). The affected genes that showed GO enrichment were classified into three categories: molecular functions (MF), cellular component (CC), and biological processes (BP) (Figure 4(b)). The genes upregulated by more than twofold had the following functions in sequential order of abundance: G-protein coupled amine receptor activity, G-protein coupled neurotransmitter receptor activity, membrane raft, membrane microdomain, membrane region, plasma membrane raft, transcription factor complex, negative regulation of apoptotic signaling pathway, hepatocellular carcinoma, and pancreatic cancer. Based on the above data, EPS inhibited NPC cells by regulating the major signaling pathways involved in the G-protein coupled receptor channel on the cell membrane and the transcription factor complex. Therefore, we used western blotting to confirm the effects of EPS.

### 3.5. The Mechanisms Underlying EPS-Induced Methuosis in NPC

The mechanisms underlying methuosis are unclear, but previous studies have identified HRAS and RAC1 GTPase as important regulators of this process [16]. To test this hypothesis, we examined the expression levels of HRAS and RAC1 in NPC cells. As shown in Figure 4(c), the levels of the senescence proteins HRAS, RAC1, RAF6, p-ERK1/2, and c-FOS were decreased in the EPS groups. Because caspase-3 is a critical mediator of apoptosis, the protein levels of caspase-3 and cleaved caspase-3 were detected by western blot analysis. There was no elevation of cleaved caspase 3 indicating that EPS-treated NPC cells did not show apoptotic signal.

**Figure 5:**
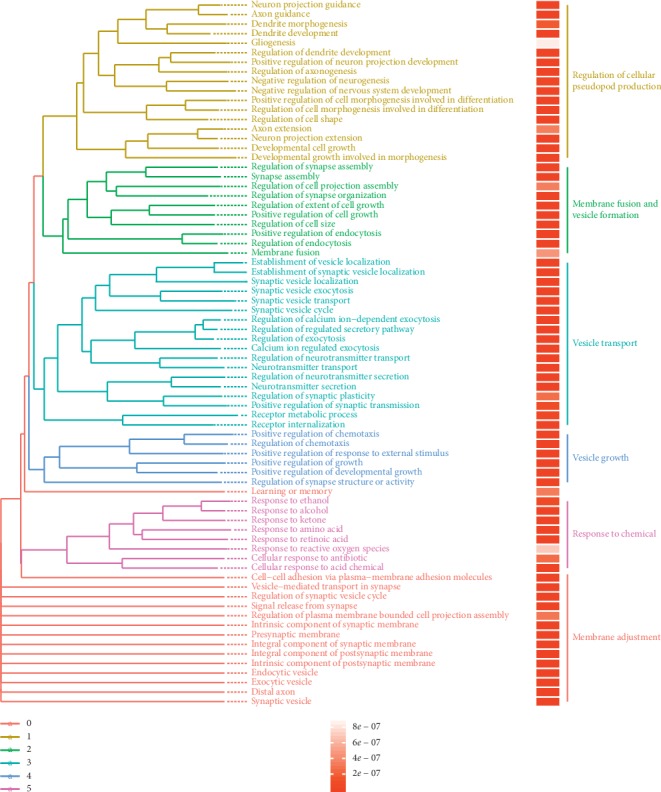
GO cluster analysis of the downstream genes of the transcription factor c-FOS during methuosis.

Furthermore, a systemic search was conducted, and a total of 1491 downstream target genes of c-FOS were defied through the Harmonizome database [17]. The GO BP analysis showed that the genes were enriched in the following pathways: (1) GO: 0060627, regulation of vesicle-mediated transport; (2) GO: s0030100, regulation of endocytosis; (3) GO: 0909003, vesicle-mediated transport in synapse; (4) GO: 00099560, synaptic membrane adhesion; (5) GO: 0009504, synaptic vesicle cycle; (6) GO: 0022604, regulation of cell morphogenesis; (7) GO: 0048259, regulation of receptor-mediated endocytosis; (8) GO: 0001101, response to acid chemical; and (9) GO: 0098693, regulation of synaptic vesicle cycle (Figures 4(d) and 4(e)). Based on the GO functional analysis, six parts were identified: cell pseudopod formation, cell membrane fusion and vesicle formation, vesicle transport, vesicle growth, stimulation of chemical substances, and membrane regulation Figure 5.

## 4. Discussion

Cells display many different forms of nonapoptotic cell death [18]. Cellular morphological features observed with TEM are commonly used to distinguish between different forms of cell death. Different types of vesicles were found in the cytoplasm in various nonapoptotic forms of cell death, such as autophagy, oncosis, and paraptosis [19]. In this study, we conducted MTT tests to select the appropriate concentration of EPS. The selected concentrations were 0, 0.25, 0.5, 1.0, 1.5, and 2.0 mg/ml. The test results showed that 1.0 mg/ml was the optimal concentration. After addition of 1.0 mg/ml EPS, we observed the formation of many vesicles, which merged with each other in the cytoplasm to form larger vesicles; furthermore, membrane rupture was observed 24 h after the EPS treatment. In this report, we identified the signaling pathways underlying a nonapoptotic form of cell death, which can be triggered by constitutive inhibition of the RAS pathway in NPC cell lines. The vacuolization in NPC cells induced by EPS is consistent with previous findings on methuosis [12].

Methuosis is a nonapoptotic form of cell death characterized by cell sprouting that induces a mutual fusion between different cells and the accumulation of vacuoles in the vesicles [20, 21]. We investigated the possible molecular mechanisms through which EPS causes cell death via methuosis and found that previous studies have shown how RAS is related to methuosis [20–22]. The results indicated that inhibition of HRAS resulted in increased methuosis. HRAS can bind to RAF6-associated vesicles and can be transported by RAF6-mediated localization of vesicles independently of the barrier protein pathway. Inactivation of RAF6 in turn affects membrane trafficking and the actin backbone, resulting in changes in cell morphology, such as vesicle production and cell sprouting [23]. Western blot results also demonstrated that EPS downregulated the protein levels of HRAS and RAF6, which are involved in methuosis of EPS-induced NPC cells.

RAC1 can induce plasma membrane protrusions to form a lamellar layered pseudopod, regulate tumor cell adhesion, and contribute to cell invasion and metastasis [24]. RAC1 also interacts with synaptojanin-2 and decreases clathrin-mediated receptor endocytosis, thereby participating in the transport of endocytic vesicles [25]. We pretreated CNE1 and CNE2 cells with ETH 1864, a specific inhibitor of RAC1 [26]. ETH 1864 could not inhibit the EPS-induced vacuolization of NPC cells. These results indicate that the methuosis induced by EPS in NPC cells does not depend on RAC1.

Studies in the literature have indicated that the Rho small G-protein is involved in important BP, such as cytoskeleton regulation, cell migration, invasion, metastasis, and cell cycle regulation, and thus can promote cell transformation and actin polymerization and remodel the extracellular matrix [27]. The Rho small G-protein family is a member of the RAS superfamily. The best-known RAS homologs, RHOA, RAC1 and CDC42, have important roles. RHOA regulates cell microfilaments and microtubules, and RAC1 and CDC42 regulate actin activity and cell adhesion, thereby promoting cytoskeletal rearrangement [28]. ERK phosphorylates the CDC42 GTPase protein, and activation of RAC1 and CDC42 promotes the formation of pseudopods at the leading edge [29]. ERK phosphorylates cytoskeletal components in the cytoplasm, such as microtubule-associated protein 1 (MAP-1), MAP-2, and MAP-4, which are involved in the regulation of cell morphology and the cytoskeletal structure [30]. Activated ERKs phosphorylate many targets, including kinases, transcription factors, and cytoskeletal proteins; these proteins include signal transducer and activator of transcription 1/3 (STAT1/3), nuclear factor-*κ*B (NF-*κ*B), the oncogene c-MYC, estrogen receptor (ESR), and the transcription factors c-JUN and c-FOS [31]. The downstream target genes of c-FOS were found to be involved in the formation of intracellular vesicles, synaptic membrane adhesion, and cell fusion during cell death.

Simultaneously, in the Cancer Genome Atlas (TCGA) database, a subgroup of head and neck squamous cell carcinoma (HNSC, samples = 508) patients with favorable clinical outcomes showed infrequent copy number alterations that were correlated with activating mutations of HRAS, RAF, RAC1, and ERK1/2 (Supplemental Figure 2). HRAS, RAF, RAC1, and ERK1/2, as targets of tumor-targeting agents, are closely related to the prevention and treatment of tumors [32].

To clarify the role of EPS in NPC cells, we conducted the series of experiments described above. However, our study has some limitations. First, EPS contains many components that could not be completely identified. Second, the downstream target genes require further investigation. Finally, the drugs that induce methuosis in cells are mainly laboratory-synthesized monomers. Synthesis of these drugs is difficult and, thus, low quantities are available. These molecules are not commercially available, which is the main reason why we could not use these drugs as positive controls.

## 5. Conclusions

In summary, we identified EPS, a product of natural plants, as a novel allergic inducer that ultimately leads to cell death. EPS can be used to explore the potential relationship between methuosis and anti-NPC effects. Our results also showed that EPS induced the formation of pseudopods, cell fusion, and accumulation of intracellular vesicles in NPC cells, leading to excess vacuoles and cell membrane rupture. Future research will focus on elucidating how EPS regulates the downstream targets of the transcription factor AP-1 and analyses of EPS in vivo. The plants that yield EPS are rich in various resources and inexpensive. We hope that further investigations of this plant and its extract-induced forms of nonapoptotic cell death will be conducted, providing new opportunities for cancer treatment.

## Data Availability

The data used to support the findings of this study are available from the corresponding author upon request.

## Supplementary Materials

Supplementary Materials The online version of this article contains Supplementary Materials. Supplemental Figure 1: HPLC fingerprint for EPS. HPLC analysis of EPS was performed with an Agilent XDB-ODS column (250 × 4.6 mm, 5 *µ*m diameter). EPS was analyzed in an Agilent 1260 series HPLC with a reversed-phase C18 column (Agilent XDB-ODS, 5 *µ*m, 4.6 × 250 mm). (a) Gallic acid standard; (b) EPS sample. (c) ellagic acid; (d) EPS sample; (e) ursolic acid; (f) EPS sample; (g) *β*-sitosterol; (h) EPS sample. Supplemental Figure 2: significantly mutated genes in head and neck squamous cell carcinoma (HNSC). The top of the picture shows the total number of mutations per megabase. The left panel indicates the frequency at which each mutation site appears in the sample; the middle panel indicates the mutation information of each gene in each sample; the color indicates the mutation type of the gene specified in the sample. Supplemental Table 1. The content of active ingredients in EPS was determined by HPLC.
