# Supplementary material for: Ethanol Extract of the Infructescence of Platycarya strobilacea Sieb. et Zucc. Induces Methuosis of Human Nasopharyngeal Carcinoma Cells
Source: Evid Based Complement Alternat Med. 2020 Apr 29;2020:2760979. doi: 10.1155/2020/2760979 (PMC7206861; doi:10.1155/2020/2760979)
Supplement: Supplementary Materials — The online version of this article contains Supplementary Materials. Supplemental Figure 1: HPLC fingerprint for EPS. HPLC analysis of EPS was performed with an Agilent XDB-ODS column (250 × 4.6 mm, 5 µm diameter). EPS was analyzed in an Agilent 1260 series HPLC with a reversed-phase C18 column (Agilent XDB-ODS, 5 µm, 4.6 × 250 mm). (a) Gallic acid standard; (b) EPS sample. (c) ellagic acid; (d) EPS sample; (e) ursolic acid; (f) EPS sample; (g) β-sitosterol; (h) EPS sample. Supplemental Figure 2: significantly mutated genes in head and neck squamous cell carcinoma (HNSC). The top of the picture shows the total number of mutations per megabase. The left panel indicates the frequency at which each mutation site appears in the sample; the middle panel indicates the mutation information of each gene in each sample; the color indicates the mutation type of the gene specified in the sample. Supplemental Table 1. The content of active ingredients in EPS was determined by HPLC. [file 2760979.f1.docx]

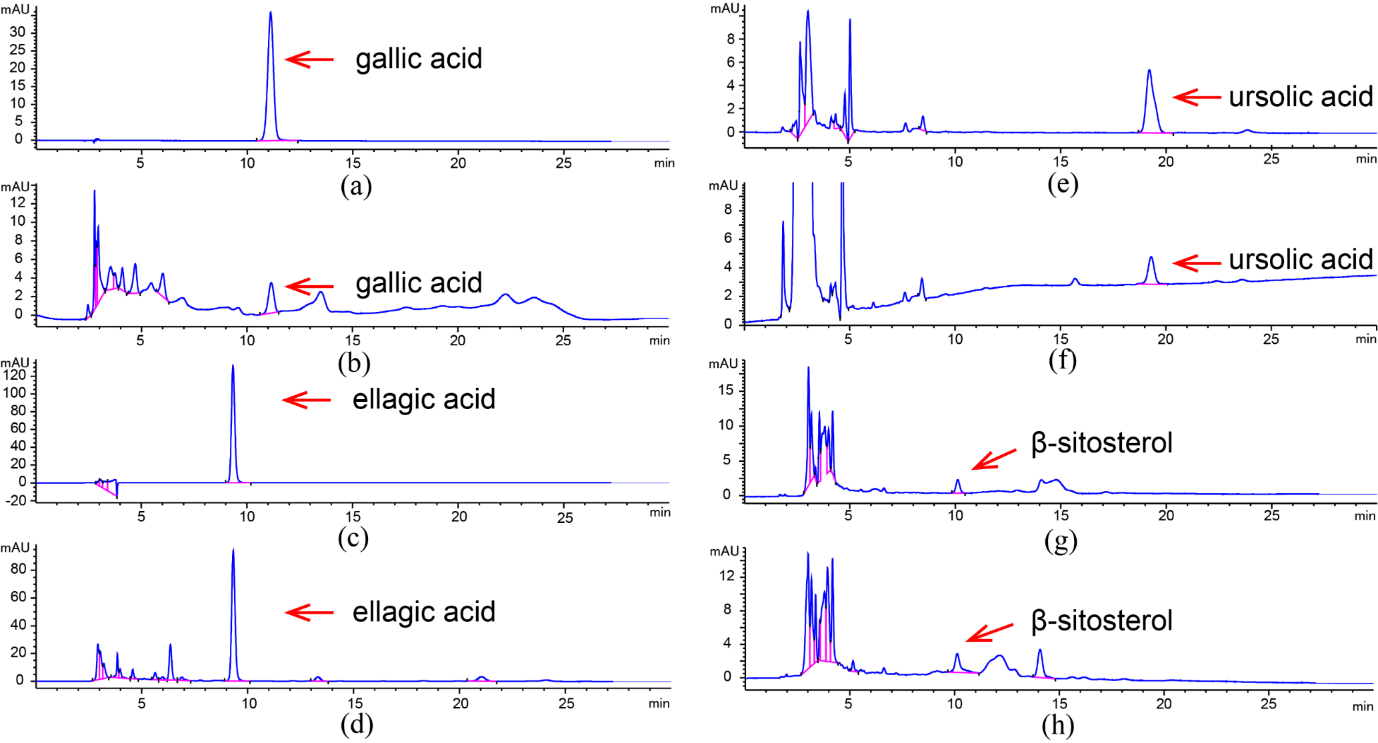


Supplemental figure 1: HPLC fingerprint for EPS. HPLC analysis of EPS was performed with an Agilent XDB-ODS column (250 × 4.6 mm, 5 μm diameter).

EPS was analyzed in an Agilent 1260 series HPLC with a reversed-phase C18 column (Agilent XDB-ODS, 5 μm, 4.6 × 250 mm).

(A) gallic acid standard; (B) EPS sample. (C) ellagic acid; (D) EPS sample. (E) ursolic acid; (F) EPS sample. (G) β-sitosterol; (H) EPS sample.


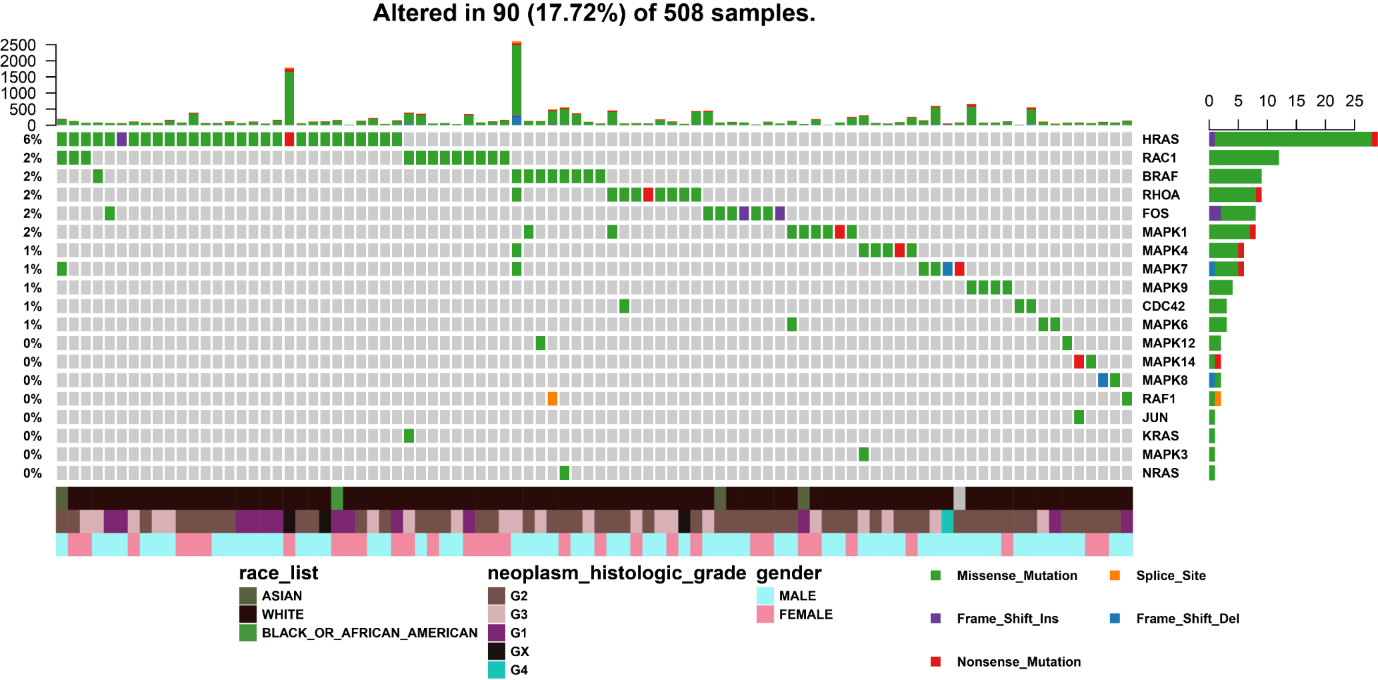


Supplemental figure 2: Significantly mutated genes in head and neck squamous cell carcinoma (HNSC). The top of the picture shows the total number of mutations per megabase. The left panel indicates the frequency at which each mutation site appears in the sample; the middle panel indicates the mutation information of each gene in each sample; the color indicates the mutation type of the gene specified in the sample.

Supplemental table 1. The content of active ingredients in EPS was determined by HPLC.

| numbering | samples | mobile phase | v:v | wavelength (nm) | column temperature (°C) |
| --- | --- | --- | --- | --- | --- |
| A | gallic acid | 0.2% phosphoric acid solution: acetonitrile | 3:97 | 270 | 30 |
| B | EPS |  |  |  |  |
| C | ellagic acid | 0.2% phosphoric acid solution: acetonitrile | 79:21 | 254 | 30 |
| D | EPS |  |  |  |  |
| E | ursolic acid | 0.1% phosphoric acid solution: acetonitrile | 25:75 | 210 | 35 |
| F | EPS |  |  |  |  |
| G | β-sitosterol | methanol | 100 | 210 | 35 |
| F | EPS |  |  |  |  |
